# A Ratiometric Fluorescent Sensor Based on Dye/Tb (III) Functionalized UiO-66 for Highly Sensitive Detection of TDGA

**DOI:** 10.3390/molecules27196543

**Published:** 2022-10-03

**Authors:** Yangchun Fan, Xin Jiang, Jie Che, Mingfeng Li, Xuejuan Zhang, Daojiang Gao, Jian Bi, Zhanglei Ning

**Affiliations:** 1 College of Chemistry and Materials Science, Sichuan Normal University, Chengdu 610068, China; 2 The Experiment Center, Shandong Police College, Ji’nan 250014, China

**Keywords:** ratiometric fluorescent sensor, urine, TDGA

## Abstract

Thiodiglycolic acid (TDGA) is a biomarker for monitoring vinyl chloride exposure. Exploring a facile, rapid and precise analysis technology to quantify TDGA is of great significance. In this research, we demonstrate a fluorescent sensor based on dual-emissive UiO-66 for TDGA detection. This ratiometric fluorescent material named C460@Tb-UiO-66-(COOH)_2_ was designed and synthesized by introducing organic dye 7-diethylamino-4-methylcoumarin (C460) and Tb^3+^ into UiO-66-(COOH)_2_. The as-obtained C460@Tb-UiO-66-(COOH)_2_ samples showed highly selective recognition, excellent anti-interference and rapid response characteristics for the recognition of TDGA. The detection limit is 0.518 mg·mL^−1^, which is much lower than the threshold of 20 mg·mL^−1^ for a healthy person. In addition, the mechanism of TDGA-induced fluorescence quenching is discussed in detail. This sensor is expected to detect TDGA content in human urine.

## 1. Introduction

Vinyl chloride (VCM) is an important industrial chemical used to produce cables, pipes and household equipment [1,2]. However, studies reveal that long-term exposure to VCM could increase the risk of cardiovascular, liver and immunocompromised diseases. VCM is classified as a Group Ι human carcinogen by the International Agency for Research on Cancer [3] and the fourth most dangerous compound by the Agency for Toxic Substances and Disease Registry [4]. However, due to the heterogeneity of individual factors, environmental monitoring does not reflect people’s actual VCM poisoning. Thiodiglycolic acid (TDGA) in human urine has been identified as a biomarker of human VCM exposure [5]. The TDGA amount excreted in human urine presents a quantitative relationship with the exposure level of VCM in the individual and it can exclude potential bias from sources including medications, nutritional supplements, creatine and ethylene oxide [4,6]. Thus, the precise determination of TDGA is necessary. At present, there a variety of analytical techniques have been used to detect TDGA, such as gas chromatography [7], isokinetic electrophoresis [8], voltammetry [9] and liquid chromatography–mass spectrometry [10]. However, these methods are difficult to use for fast and real-time monitoring because of their complicated sample pretreatment, long processing time and inadequate detection limits [11,12]. Therefore, a simple and precise method for the rapid identification of TDGA levels is highly desirable.

In recent years, fluorescence detection has received great attention owing to its facile operation, low instrument cost, fast response and high sensitivity [13,14]. Luminescent metal-organic frameworks (LMOFs) based on metal ions and organic linkers have become attractive fluorescence materials [15,16]. Owing to their permanent porosity, large specific surface area and strong emissions, this class of materials have been widely used in the sensing field, such as metal ions [17,18], volatile organic compounds [19], and anions [20,21] or as a temperature sensor [22]. Among them, UiO-66 has emerged as a novel fluorescent sensor with ultra-high chemical stability, thermodynamic stability and good biocompatibility [23,24]. However, the unsatisfactory luminescence properties limit its further application in the field of fluorescence. It was found that excellent luminescent UiO-66 hybrid materials could be obtained through the iso-expansion of ligands and post-synthesis modification. In particular, Ln-UiO-66, obtained by introducing rare earth ions, has attracted wide attention due to its high luminescent quantum yield [25,26]. However, most of Ln-UiO-66 is based on a single emission, which may be greatly interfered with by the concentration, the background of the fluorescence signal and the environmental conditions. In comparison, ratiometric fluorescence detection can be used as a desired method to measure the target by quantitative detection of the fluorescence response based on two separate fluorescence emission wavelengths, in which one emission peak acts as a reference and the other one acts as a signal response unit [13,27]. Since this measured fluorescence excludes the background interference, reduces the extrapolation errors and environmental factors, the ratiometric fluorescent sensor can greatly enhance the sensitivity of the detection system. However, research into UiO-66-based ratiometric fluorescent sensors is very rare. This is mainly caused by the deficiency of binding sites in the UiO-66 matrix, which prevents lanthanide ions or other guest molecules form a stable structure with UiO-66. Therefore, it is imperative to develop and design functionalized UiO-66-based ratiometric fluorescent sensors for effective sensing of analytes.

In this work, UiO-66-(COOH)_2_ with isomeric ligand extension was synthesized firstly. Then, a new ratiometric fluorescent material C460@Tb-UiO-66-(COOH)_2_ was designed and synthesized by simultaneous introducing the organic dye (7-diethylamino-4-methylcoumarin, C460) and Tb^3+^ into the pores of UiO-66-(COOH)_2_. It was found that the C460@Tb-UiO-66-(COOH)_2_ samples with double blue-green emission have the advantages of excellent selectivity, high sensitivity, and rapid response for detection Thiodiglycolic acid (TDGA) in urine. In addition, fluorescent test paper was successfully prepared for the rapid detection of TDGA. The color changes visible to the naked eye were presented. This functionalized UiO-66 material is expected to be an ideal sensor for TDGA detection.

## 2. Experimental Details

### 2.1. Chemicals and Instruments

All chemicals used in this experiment were analytically pure and used without purification. All measurements were performed at room temperature.

### 2.2. Synthesis of UiO-66-(COOH)_2_

UiO-66-(COOH)_2_ was synthesized using a hydrothermal method with some modifications [28]. Zirconium tetrachloride (ZrCl_4_, 1.06 g), 1,2,4,5-benzenetetracarboxylic (H_4_btec, 0.126 g) and terephthalic acid (TPA, 0.4989 g) were dissolved into 50 mL DMF and stirring for 15 min at room temperature. Then, 5 mL acetic acid was added to adjust the crystal size and morphology. After that, the mixture was transferred to a Teflon reactor and kept at 160 °C for 24 h. Then, the white solid sample was centrifuged at 4000 rpm for 4 min, and washed with distilled water and ethanol three times, respectively. To remove residual DMF from the samples, the solids were suspended in 30 mL of acetone for one week, during which the acetone solution was changed daily. Finally, the product was recovered by centrifugation and dried at 70 °C.

### 2.3. Synthesis of Tb-UiO-66-(COOH)_2_ and C460@Tb-UiO-66-(COOH)_2_

Tb-UiO-66-(COOH)_2_ was synthesized with a simple post-synthesis method: 200 mg UiO-66-(COOH)_2_ were dispersed in 20 mL of ethanol of Tb(NO_3_)_3_·6H_2_O (0.02 M) and the mixture was stirred for 24 h at room temperature. After centrifugation, washed with ethanol, the products were dried at 70 °C for 24 h.

The synthesis of C460@Tb-UiO-66-(COOH)_2_ is identical to Tb-UiO-66-(COOH)_2_, except for the ethanol solution change to the solution of a different concentration of C460.

### 2.4. Determination of the Content of Dyes in C460@Tb-UiO-66-(COOH)_2_ Samples

The luminescence intensity at 445 nm was measured for different concentrations of C460, and the measurements were repeated five times for each concentration. The standard curve of the measured concentrations and intensities was obtained.

The 20 mg C460@Tb-UiO-66-(COOH)_2_ sample was dissolved in 5 mL ethanol solution, and 30 μL HCl (12 mol·L^−1^) was added to form a transparent solution in order to exclude the fluorescence effect of the material itself. After diluting the solution with ethanol 10 times, the characteristic emission intensity of C460 in the material was determined.

### 2.5. C460@Tb-UiO-66-(COOH)_2_ Materials were Used for TDGA Testing Experiments

The 2 mg C460@Tb-UiO-66-(COOH)_2_ sample was weighed and dissolved in distilled water (3 mL) for 30 min after ultrasonic treatment. Then, 1 mL of 0.02 mol·L^−1^ urine component solutions (NH_4_Cl, NaCl, KCl, glucose (Glu), creatine (Cre), creatinine, urea, thiodiglycolic acid) were added to the suspension. After that, the suspension was analyzed by fluorescence.

### 2.6. Preparation of C460@Tb-UiO-66-(COOH)_2_ Fluorescent Test Paper and Urine Composition Detection Experiment

The preparation method of the test paper was as follows: First, the test paper was processed into strips of the same size and soaked in C460@Tb-UiO-66-(COOH)_2_ suspension prepared for 5 min. After soaking, the test paper was removed and allowed to dry in the natural environment.

The specific detection method was as follows: A total of 50 μL TDGA was removed with a pipette gun and added to the test paper attached C460@Tb-UiO-66-(COOH)_2_. After 1 min, the test paper was placed under a UV lamp and the color change of the test paper was observed at 254 nm as excitation wavelength.

## 3. Results and Discussion

### 3.1. Characterizations of C460@Tb-UiO-66-(COOH)_2_

**Figure 1:**
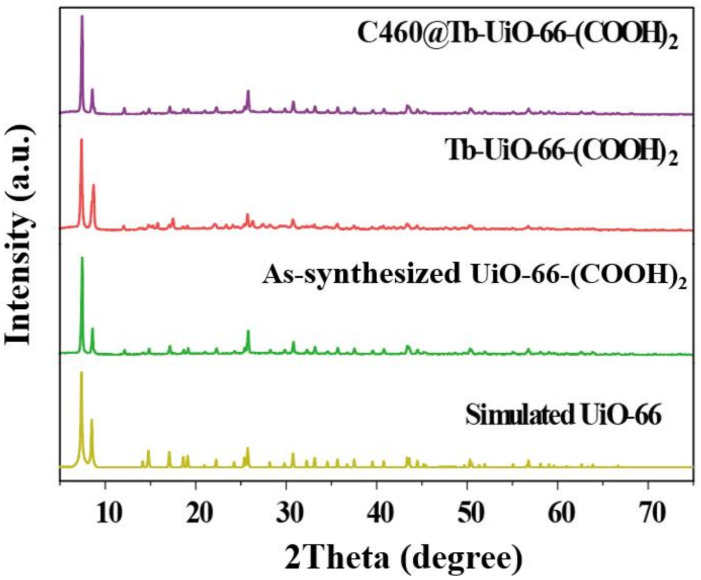
XRD patterns of simulated UiO-66, as-synthesized UiO-66-(COOH)_2_, Tb-UiO-66-(COOH)_2_, C460@Tb-UiO-66-(COOH)_2_.

**Figure 2:**
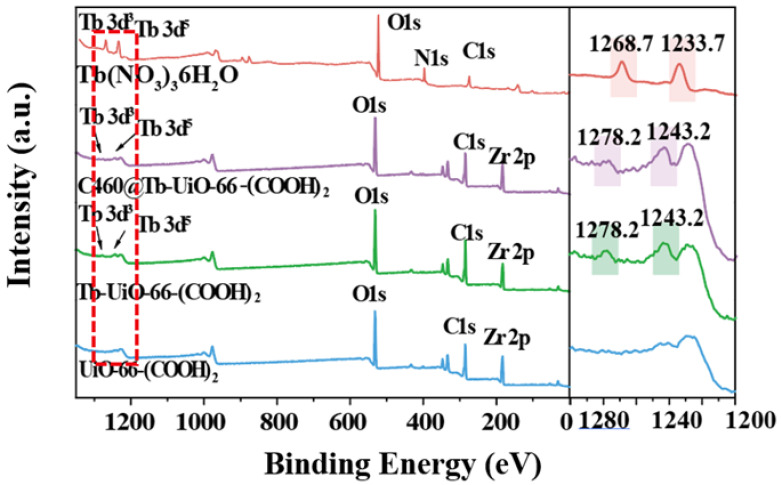
XPS spectra analysis of Tb(NO_3_)_3_·6H_2_O, UiO-66-(COOH)_2_, Tb-UiO-66-(COOH)_2_, C460@Tb-UiO-66-(COOH)_2_.

Firstly, the X-ray diffraction (XRD) analysis was performed (Figure 1). It can be observed that the XRD pattern of UiO-66-(COOH)_2_ is similar to the simulated UiO-66, indicating that the pure phase of UiO-66-(COOH)_2_ has been successfully synthesized by the hydrothermal method. After the modification of Tb^3+^ and C460, the crystalline phase of C640@Tb-UiO-66-(COOH)_2_ is also consistent with the UiO-66-(COOH)_2_ nanocrystalline, which means the post-synthetic modification process did not change the structure of the material [29]. The XPS spectra of the as-obtained UiO-66-(COOH)_2_, Tb-UiO-66-(COOH)_2_ and C460@Tb-UiO-66-(COOH)_2_ were tested and the results are shown in Figure 2. From the full spectrum, peaks of 3d^3^ and 3d^5^ Tb appeared in the energy spectra of both Tb-UiO-66-(COOH)_2_ and C460@Tb-UiO-66-(COOH)_2_, which could be clearly observed from the energy spectra of the enlarged 1200–1300 eV, indicating that Tb^3+^ was successfully introduced into the UiO-66-(COOH)_2_ material. Moreover, the successful coordination of Tb^3+^ to the free -COO^−^ chelating sites in the UiO-66-(COOH)_2_ can also be confirmed by the binding energy of Tb^3+^ 3d. The peak position of Tb^3+^ 3d^3^ and 3d^5^ of Tb-UiO-66-(COOH)_2_ and C460@Tb-UiO-66-(COOH)_2_ exhibits higher binding energy (1278.2 and 1243.2 eV) compared to that of Tb(NO_3_)_3_·6H_2_O (1268.7 and 1233.7 eV). The varied binding energy indicates that Tb^3+^ was successfully introduced into the material and coordinated with the free carboxyl group of the ligand [30].

Figure S1 shows the SEM and elemental mapping of C460@Tb-UiO-66-(COOH)_2_. The particle size of the C460@Tb-UiO-66-(COOH)_2_ sample is about 80~100 nm. The morphology is similar to that of the reported UiO-66 [31], demonstrating that the introduction of C460 and Tb^3+^ does not change the revealed morphology of the material. The sample mapping shows that four elements C, O, Zr and Tb were evenly dispersed in the UiO-66-(COOH)_2_ material, indicating that C460 and Tb^3+^ were successfully introduced into the framework of UiO-66-(COOH)_2_. Moreover, the energy dispersive X-ray analysis (EDX) spectra (Figure S2) is another proof of the successful introduction of Tb^3+^. Detailed data are presented in Table S1, the elemental content of C, O, Zr, Tb and Au are 71.32%, 22.7%, 4.64%, 0.31% and 1.03%, respectively, and the content ratio of Tb and Zr is about 30%.

The N_2_ adsorption of the sample was further measured. As exhibited in Figure S3, the specific surface area and pore volume of UiO-66-(COOH)_2_ were 449.8695 m^2^/g and 0.35 cm^3^/g, respectively, demonstrating that UiO-66-(COOH)_2_ is a three-dimensional (3D) material with a high specific surface area. The adsorption average pore diameter (4 V/A) of Barret–Joyner–Halenda was 108.026 Å, indicating that its porous structure could provide lattice space for guest molecules [32]. After adding C460 and Tb^3+^, the specific surface area and pore volume of C460@Tb-UiO-66-(COOH)_2_ are 357.0713 m^2^/g and 0.28 cm^3^/g, which decrease by 20.63% and 20.00%, respectively. The results further prove that Tb^3+^ and C460 have been introduced into the material [33].

### 3.2. Fluorescence Property of C460@Tb-UiO-66-(COOH)_2_

**Figure 3:**
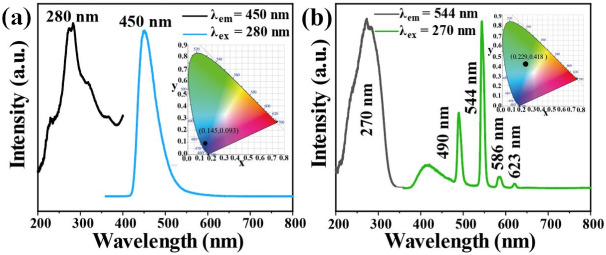
(**a**) Room temperature excitation and emission spectra of C460 and (**b**) Tb-UiO-66-(COOH)_2_.

The photoluminescence properties of Tb-UiO-66-(COOH)_2_ and C460 were measured at room temperature. From the excitation and emission spectra of C460 (Figure 3a), it can be observed that the excitation spectrum of C460 shows a wide excitation peak in the range of 200–400 nm and the strongest absorption peak is located at 280 nm. Using 280 nm as the excitation wavelength, a strong emission peak appears at 450 nm. The CIE coordinate is calculated to be (0.145, 0.093), which is located in the blue region, indicating that C460 is an excellent blue luminescent material. Similarly, Figure 3b shows the excitation and emission spectra of Tb@UiO-66-(COOH)_2_. The excitation spectra obtained at 544 nm show obvious absorption peaks in the range of 200–350 nm, which are attributed to the π–π* transition of the ligand and the electronic transition of Tb^3+^ ions from the ground state (S_0_) to the excited state (S_1_). The wide excitation peak is more conducive to an energy transfer from the ligand to Tb^3+^. Therefore, at 270 nm excitation, the emission spectrum of Tb-UiO-66-(COOH)_2_ clearly shows the characteristic peak of Tb^3+^ from ^5^D_4_→^7^F_J_ (J = 6, 5, 4, 3) at 490, 544, 586 and 623 nm, respectively [19]. In addition, a wide and weak emission peak based on ligand luminescence was also observed in the 380 to 480 nm range, which is much smaller than the characteristic terbium emission, indicating that the “antenna effect” of ligands and terbium ions is effective. Tb-UiO-66-(COOH)_2_ shows a strong green emission, which is consistent with the calculated CIE coordinate (0.229, 0.418).

**Figure 4:**
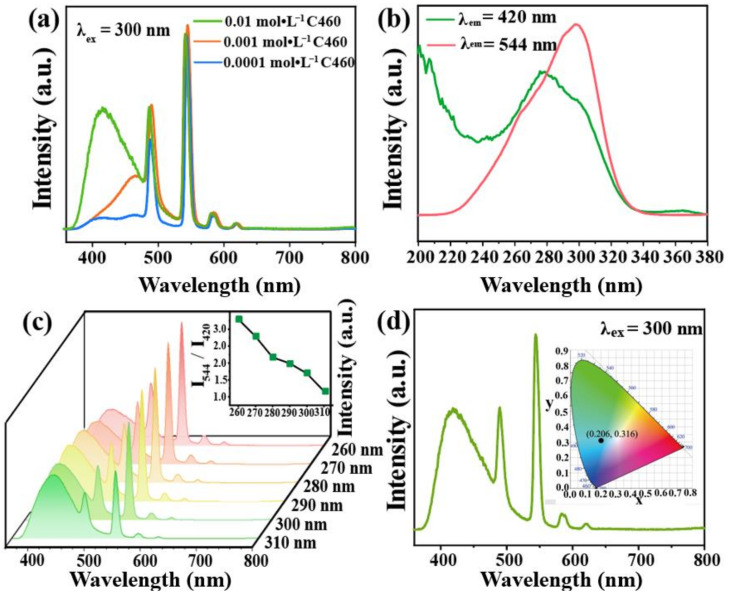
(**a**) Emission spectra of C460@Tb-UiO-66-(COOH)_2_ with different initial concentrations of C460; (**b**) Excitation spectra of C460@Tb-UiO-66-(COOH)_2_ monitored at 420 nm and 544 nm, respectively; (**c**) Emission of C460@Tb-UiO-66-(COOH)_2_ with the excitation wavelength from 260–310 nm; (**d**) Room temperature emission spectra of C460@Tb-UiO-66-(COOH)_2_, inset is CIE of chromaticity diagram of C460@Tb-UiO-66-(COOH)_2_.

To prepare the dual-emission material C460@Tb-UiO-66-(COOH)_2_, different concentrations of C460 were added to Tb-UiO-66-(COOH)_2_. The emission spectra are shown in Figure 4a. It is found that the samples prepared at 0.01 mol·L^−1^ C460 show the characteristic emissions of C460 and Tb^3+^, and the ratio of intensity is close to 1:2. This concentration was chosen for subsequent experiments. To accurately determine the loading content of C460, the emission spectra of a series of C460 solutions with different concentrations and C460@Tb-UiO-66-(COOH)_2_ (0.01 M C_460_) were recorded (Figure S4). Based on the linear fitting equation, the content of dyes in the C460@Tb-UiO-66-(COOH)_2_ samples was determined to be 1.38%.

In order to obtain the optimal excitation wavelength of the material, the excitation spectra of C460@Tb-UiO-66-(COOH)_2_ were performed with the monitoring wavelength of 420 nm and 544 nm. The maximum excitation band of C460@Tb-UiO-66-(COOH)_2_ appeared at 270 nm and 300 nm (Figure 4b), respectively. The emission spectra are recorded under different excitation wavelength in the range of 260–310 nm. As seen in Figure 4c, all of the emission spectra include the C460 emission line and the Tb-UiO-66-(COOH) emission line. However, the emission peak of C460 at 420 nm is gradually enhanced, while that of the terbium ion at 544 nm is decreased, successively. Considering the emission intensity and the ratio of green/blue emission (I_544_/I_420_), we chose 300 nm as the excitation wavelength for the next experimental condition. In addition, the CIE chromaticity diagram is located at (0.206, 0.316), showing a blue-green light as shown in Figure 4d. In summary, we chose a dye concentration of 0.01 M and an optimal excitation wavelength of 300 nm as the optional experimental conditions.

### 3.3. Ratio Fluorescent Sensing Behavior of C460@Tb-UiO-66-(COOH)_2_ towards TDGA

**Figure 5:**
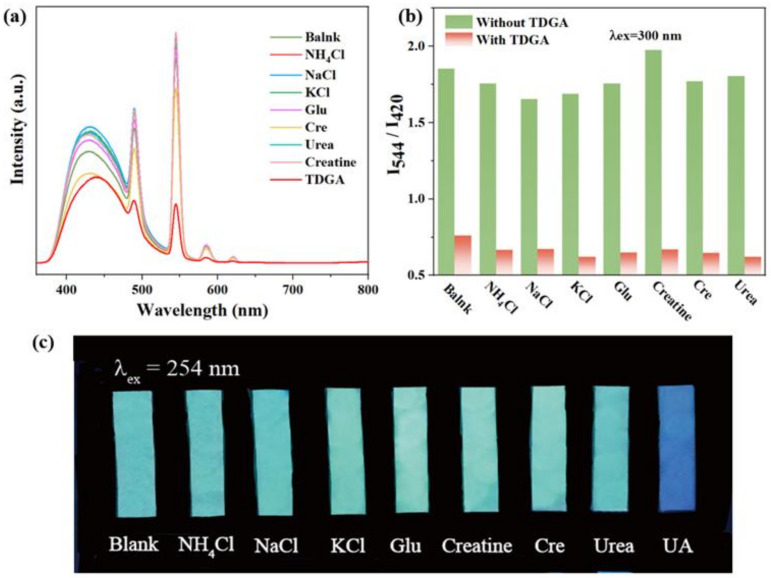
(**a**) Emission spectra of C460@Tb-UiO-66-(COOH)_2_ with various constituents in human urine; (**b**) Anti-interference of C460@Tb-UiO-66-(COOH)_2_ for detection of TDGA in the presence of different urine chemicals; (**c**) The photographs of C460@Tb-UiO-66-(COOH)_2_ having various urine components under 254 nm UV light irradiation.

Considering the pH value in human urine is 4.5–8, pH tolerance is essential to the ideal fluorescence sensor. The emission spectra of C460@Tb-UiO-66-(COOH)_2_ were firstly investigated at different pH values (Figure S5). The fluorescence intensity at 420 nm and 544 nm remained essentially unchanged at pH values from 3 to 9, which confirms its outstanding pH stability. In view of the excellent ratiometric fluorescence and stable structure, we designed the composite C460@Tb-UiO-66-(COOH)_2_ as a fluorescent sensor and researched its potential application for detecting common urinary constituents. Figure 5a gives the fluorescence influence of eight typical urine components on the target sensor C460@Tb-UiO-66-(COOH)_2_, including NH_4_Cl, NaCl, KCl, glucose (Glu), creatine (Cre), creatinine, urea and thiodiglycolic acid (TDGA). The selectivity experiments are measured under the same conditions. From the emission spectrum, it can be seen that the intensity of the different urine components to C460@Tb-UiO-66-(COOH)_2_ is discrepant. Among them, the most remarkably prominent is that of TDGA with C460@Tb-UiO-66-(COOH)_2_. The fluorescence intensity of Tb^3+^ ion at 544 nm decreased significantly, while the fluorescence intensity of C460 at 420 nm decreased slightly. It can be clearly found from the emission ratio (I_544_/I_420_) of C460@Tb-UiO-66-(COOH)_2_ treated by TDGA that it is reduced greatly, which is about 0.15-fold less than the blank solution and other urine components. Similarly, the same result can be further confirmed in Figure 5c. Under 254 nm UV light irradiation, the test paper of C460@Tb-UIO-66-(COOH)_2_ with TDGA exhibits a pale blue color, in contrast to the ones with other urine components that show a blue-green color. These results indicate that this fluorescent sensor has a high selectivity for TDGA detection in urine. In addition, the ability of anti-interference of sensors for other urine components in the detection of TDGA was checked. As shown in Figure 5b, the intensity ratio (I_544_/I_420_) sharply decreased after introducing TDGA with coexistent interfering components. This result reveals that the response of C460@Tb-UiO-66-(COOH)_2_ is not influenced by the additions of other urine components, exhibiting good anti-interference capability and high selectivity towards TDGA [34]. Therefore, the C460@Tb-UiO-66-(COOH)_2_ is considered as a promising fluorescence sensor for TDGA.

**Figure 6:**
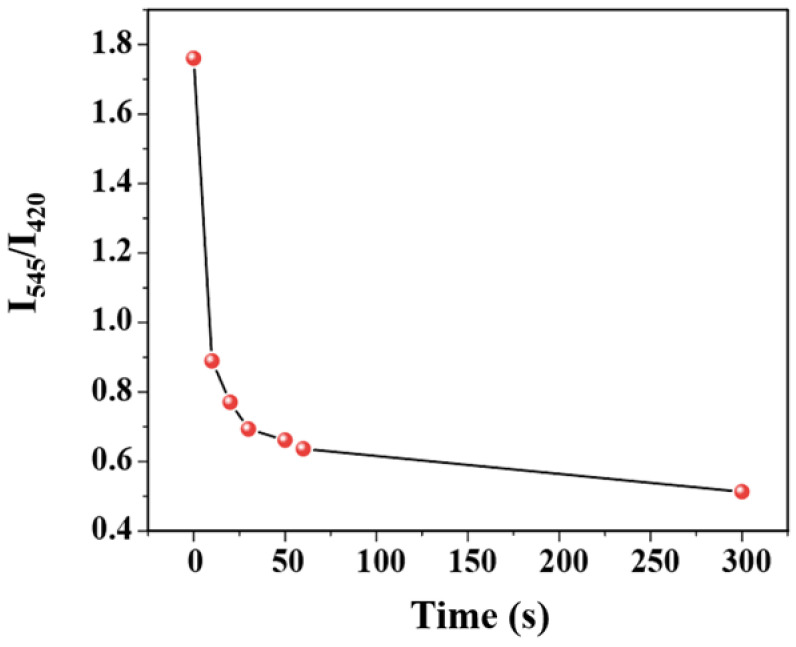
Function relationship between I_544_/I_420_ ratio and immersion time after entering TDGA.

The time–response characteristic of the C460@Tb-UiO-66-(COOH)_2_ sensor toward TDGA has been investigated. From the function relationship between I_544_/I_420_ and immersion time after entering TDGA (Figure 6), it can be seen that after the introduction of TDGA, the ratio of luminescence intensity (I_544_/I_420_) of C460@Tb-UiO-66-(COOH)_2_ shows a sharp reduction after the addition of TDGA for 10 s and levels off in 1 min. Compared with previously reported literature, the C460@Tb-UiO-66-(COOH)_2_ material exhibits excellent sensing performance [35].

**Figure 7:**
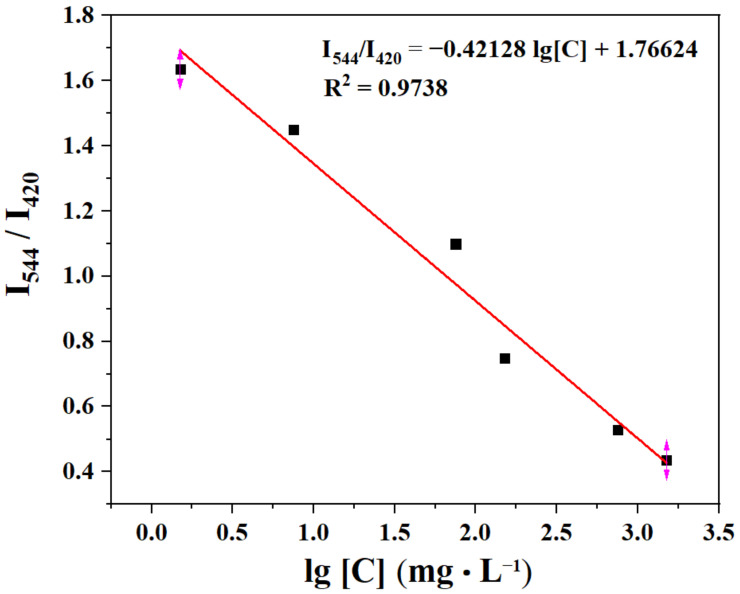
The plot of I_544_/I_420_ vs. the TDGA concentration.

Sensitivity is a major factor in determining the application of a sensor in real-life applications. Concentration-dependent experiments were further investigated to evaluate the sensitivity. The sensitivity experiment was performed by measuring the corresponding fluorescence intensity with different concentrations of the TDGA in the aqueous solutions. There is a linear relationship between them in the concentration range from 0 to 0.01 M (Figure 7). The linear correlation fitting curve is I_544_/I_420_ = −0.42128 lg[*C*] + 1.76624 and the correlation coefficient (R^2^) is 0.9738, where [*C*] presents the concentration of TDGA. According to three IUPAC criteria, the limit of detection (LOD) is calculated by the following two equations:

(1) $$S_{b}^{}=\sqrt{\frac{\sum {\left(F_{0}^{}-F_{1}^{}\right)}_{}^{2}}{N-1}}$$

(2) $$\mathrm{LOD}=3S_{b}/S$$

where *S*_b_ is the standard deviation; *N* is the number of blank (*n* = 21); *F*_0_ is the I_544 nm_/I_420 nm_ of C460@Tb-UiO-66-(COOH)_2_ in water; *F*_1_ is the mean of *F*_0_; *S* is the slope of the linear relationship in Figure 7. The LOD was estimated to be 0.518 mg·mL^−1^, which is far below the threshold value of 20 mg·mL^−1^ in healthy individuals [6,36].

### 3.4. Sensing Mechanisms

The mechanism for detecting TDGA by C460@Tb-UiO-66-(COOH)_2_ has been further investigated in detail. Generally, the mechanism for the detection can be attributed to three reasons [30]: (1) the collapse of the framework of C460@Tb-UiO-66-(COOH)_2_; (2) the interaction between the lanthanide ions and TDGA; (3) the interaction between ligands and TDGA. To reveal the possible sensing mechanisms, XRD analysis was first carried out (Figure S6a). After adding TDGA, the main peaks of C460@Tb-UiO-66-(COOH)_2_ are the same as those of the original one, indicating structural collapse or reorganization could be excluded.

In the abstract, the luminescence efficiency of LMOFs depends on the energy transfer between the metal and organic ligand [37]. In general, whether the energy resonance transfer between the fluorescence sensor and the analyte could occur effectively depends on the degree of overlap between the UV absorption peak of the analyte and the excitation wavelength of the luminescence sensor. Therefore, the corresponding UV–vis spectra have been explored and the results are shown in Figure S6b. There is no overlap between the UV absorption wavelength of TDGA and the excitation spectrum of C460@Tb-UiO-66-(COOH)_2_, revealing that the energy transfer between the ligand and TDGA is inefficient and it can-not actually affect the luminescence efficiency of the Tb^3+^ [38].

**Figure 8:**
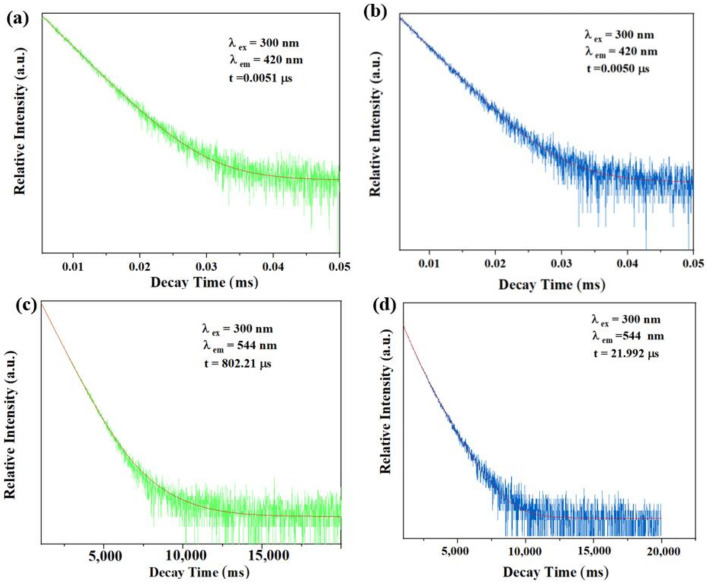
The fluorescence lifetime of the sample at 420 nm in the absence (**a**) and presence (**b**) of TDGA; The fluorescence lifetime of the sample at 544 nm in the absence (**c**) and presence (**d**) of TDGA.

Fluorescence lifetime is a very important parameter to judge the fluorescence quenching mechanism, which could usually reflect whether the quenching effect of TDGA to C460@Tb-UiO-66-(COOH)_2_ is based on a static mechanism or dynamic mechanism. Currently, the static mechanism is due to the formation of non-emitting intermediates between the fluorophore and the quenching agent, while the dynamic quenching is attributed to the collision between the excited fluorophore and the quenching agent [39]. Therefore, we further tested the fluorescence lifetime of the samples. As shown in Figure 8, after adding TDGA, the fluorescence lifetime of C460@Tb-UiO-66-(COOH)_2_ monitored at 420 nm is almost unchanged (from 0.0051 μs to 0.0050 μs). However, the fluorescence lifetime of C460@Tb-UiO-66-(COOH)_2_ monitored at 544 nm is significantly reduced from 802.2 μs to 21.992 μs. It indicates that the fluorescence quenching in this study is mainly caused by the interaction between terbium ion and TDGA, which belongs to dynamic quenching.

## 4. Conclusions

In conclusion, a ratiometric fluorescent probe C460@Tb-UiO-66-(COOH)_2_ with blue-green dual emission has been fabricated and developed as an excellent fluorescence analysis probe for the sensing of TDGA. Aqueous suspensions of C460 and Tb-UiO-66-(COOH)_2_ simultaneously generate the characteristic emission of lanthanide ions and dye with a single excitation mode. It was found that C460@Tb-UiO-66-(COOH)_2_ can be used as a fluorescent probe for TDGA recognition. This probe has the advantages of excellent selectivity, good sensitivity and fast response. The calculated detection limit is much lower than the threshold value in healthy subjects. In addition, a portable C460@Tb-UiO-66-(COOH)_2_ test paper was prepared. Based on this, C460@Tb-UiO-66-(COOH)_2_ is expected to be a fluorescent sensor to detect TDGA in urine.

## Data Availability

Not applicable.

## Supplementary Materials

The following supporting information can be downloaded at: https://www.mdpi.com/article/10.3390/molecules27196543/s1, Figure S1. The SEM and element mapping of C460@Tb-UiO-66-(COOH)_2_; Figure S2. EDX spectrum of the as-obtained C460@Tb- UiO-66-(COOH)_2_; Figure S3. The N_2_ adsorption isotherms of UiO-66-(COOH)_2_ and C460@Tb-UiO-66-(COOH)_2_ after heat-treatment; Figure S4. (a) Emission spectra of C460 in ethanol solution with different concentrations. (b) The fitted curve between the intensity and the concentration; Figure S5. (a) Fluorescence spectra of C460@Tb-UiO-66-(COOH)_2_ at different pH values. (b) Fluorescence intensity of C460@Tb-UiO-66-(COOH)_2_ at 545 nm and 420 nm at different pH values; Figure S6. (a) XRD patterns of solid-state C460@Tb-UiO-66-(COOH)_2_ and C460@Tb-UiO-66-(COOH)_2_ after, respectively, soaking in the aqueous solutions of TDGA for 24 h. (b) UV–visible absorption spectra of various constituent in human urine and excitation spectra of C460@Tb-UiO-66-(COOH)_2_; Table S1 C460@Tb-UiO-66-(COOH)_2_ determined by energy dispersive analysis by X-rays (EDX).

## Sample Availability

Samples of the compounds are available from the authors.
