# Supplementary material for: A Ratiometric Fluorescent Sensor Based on Dye/Tb (III) Functionalized UiO-66 for Highly Sensitive Detection of TDGA
Source: Molecules. 2022 Oct 3;27(19):6543. doi: 10.3390/molecules27196543 (PMC9570906; doi:10.3390/molecules27196543)
Supplement: Supplementary file 1 [file molecules-27-06543-s001.zip › molecules-1947375-supplementary.pdf]

Supporting Information

A ratiometric fluorescent sensor based on dye/ Tb (Ⅲ) functionalized UiO-66 for highly sensitive detection of TDGA

Yangchun Fan [1], Xin Jiang[1], Jie Che[1], Mingfeng Li[1], Xuejuan Zhang[2], Daojiang Gao[1], Jian Bi[1], Zhanglei Ning[1,] *

1 College of Chemistry and Materials Science, Sichuan Normal University, Chengdu 610068, China
2 The Experiment Center, Shandong Police College, Ji'nan 250014, China
* Correspondence: zlning@sicnu.edu.cn; Tel.: +86-28-84760802

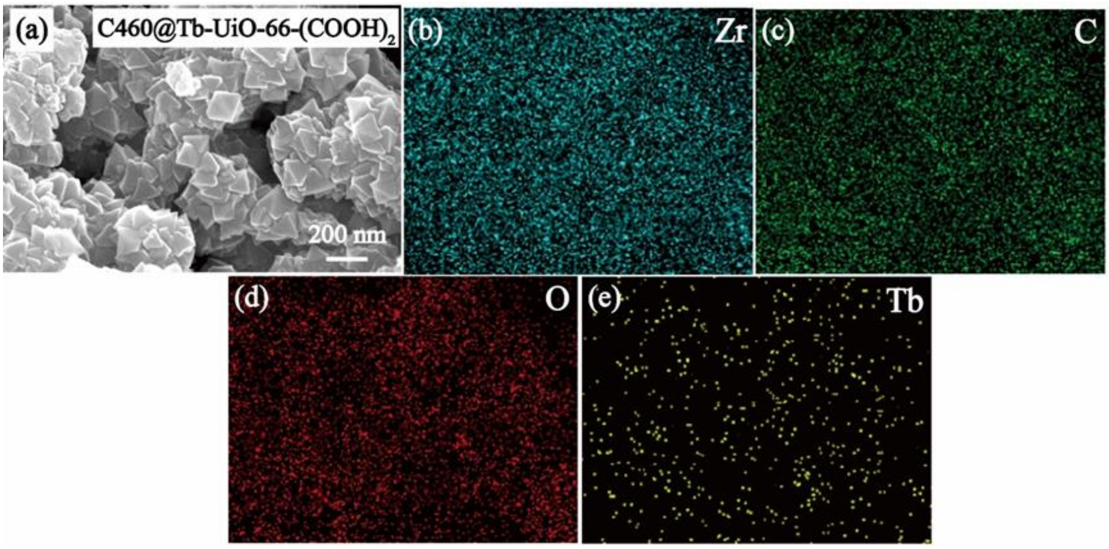

Figure S1 The SEM and element mapping of C460@Tb-UiO-66-(COOH)$_2$.

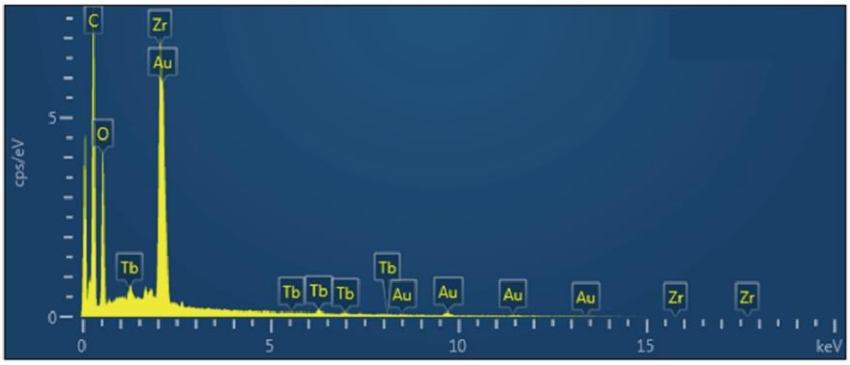

Figure S2 EDX spectrum of the as-obtained C460@Tb- UiO-66-$(COOH)_2$.

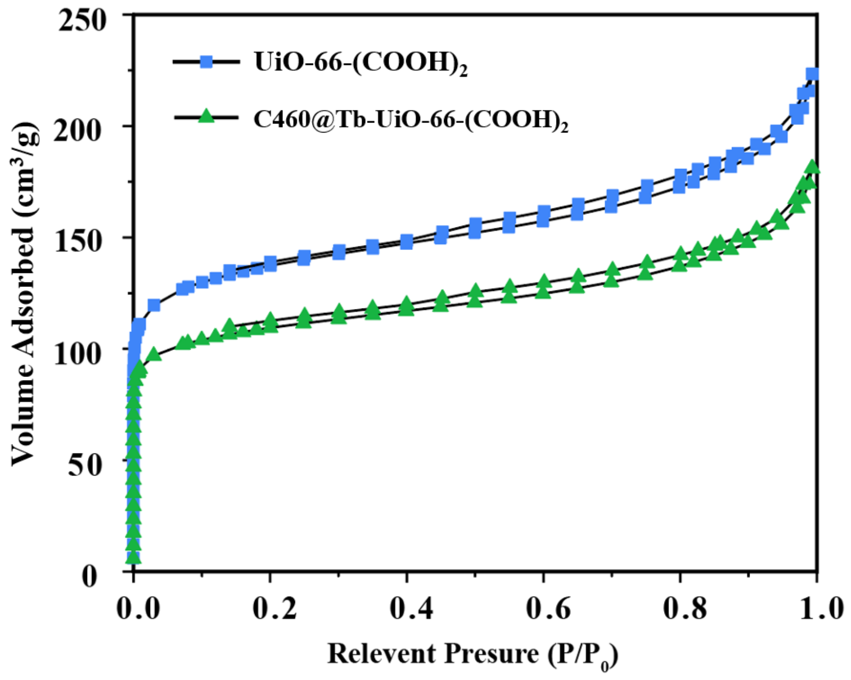

Figure S3 The N_2 adsorption isotherms of UiO-66-(COOH)$_2$ and C460@Tb-UiO-66-(COOH)$_2$ after heat-treatment.

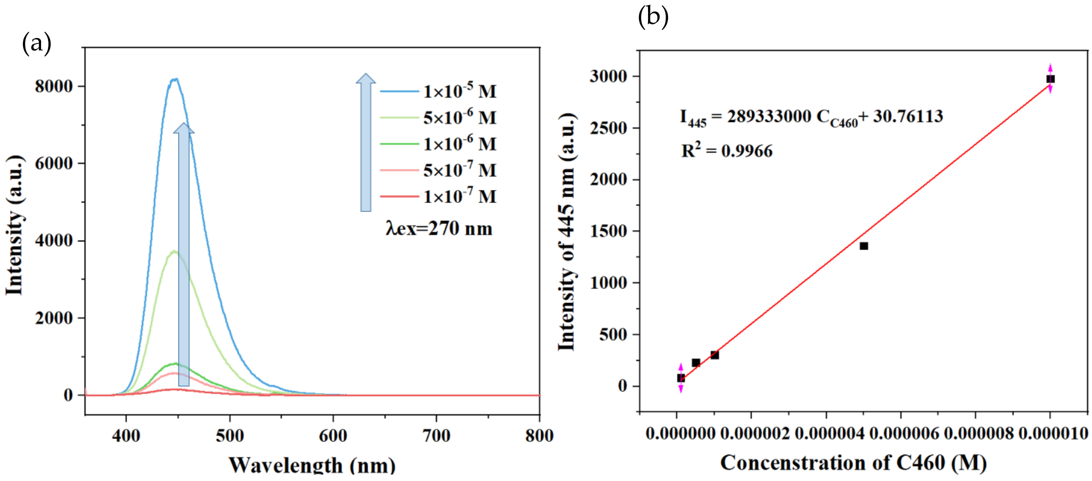

Figure S4 (a) Emission spectra of C460 in ethanol solution with different concentrations. (b) The fitted curve between the intensity and the concentration.

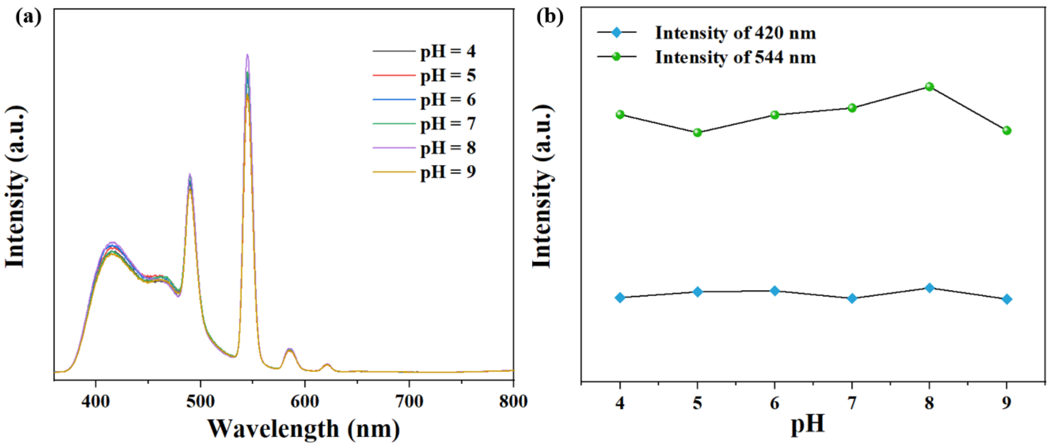

Figure S5 (a) Fluorescence spectra of C460@Tb-UiO-66-$(COOH)_2$ at different pH values; (b) Fluorescence intensity of C460@Tb-UiO-66-$(COOH)_2$ at 545 nm and 420 nm at different pH values.

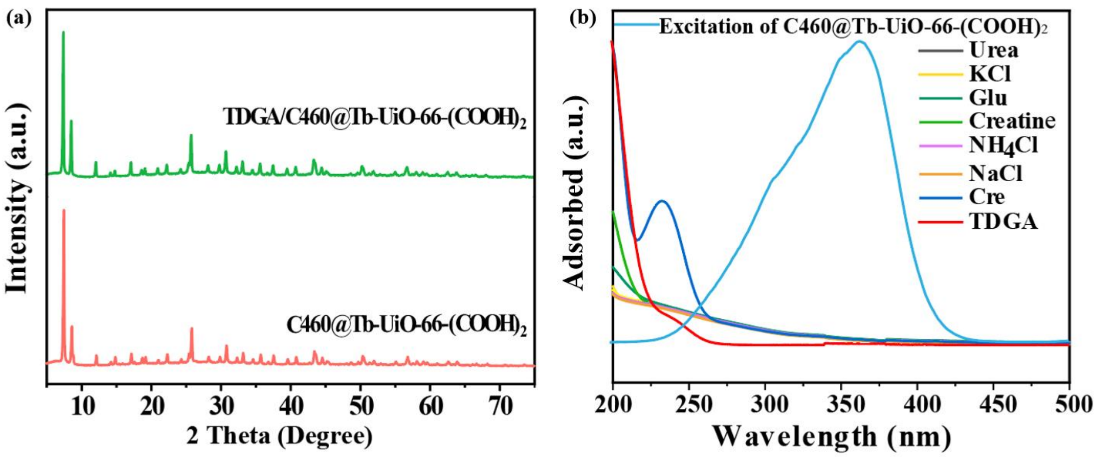

Figure S6 (a) XRD patterns of solid-state C460@Tb-UiO-66-$(COOH)_2$ and C460@Tb-UiO-66-$(COOH)_2$ after respectively soaking in the aqueous solutions of TDGA for 24 h; (b)UV-visible absorption spectra of various constituent in human urine and Excitation spectra of C460@Tb-UiO-66-$(COOH)_2$.

Table S1 C460@Tb-UiO-66-$(COOH)_2$ determined by Energy dispersive analysis by X-rays (EDX).

Element	C	O	Zr	Tb	Au
Atomic %	71.32	22.70	4.64	0.31	1.03
